# Exploring the inter-professional experiences of student nurses and midwives to facilitate better outcomes for mothers with a learning disability in maternity services: a qualitative case study

**DOI:** 10.1177/17449871251328891

**Published:** 2026-06-02

**Authors:** Angela Ridley, Amy Swift, Amy Cole

**Affiliations:** Assistant Professor/Head of Subject Nursing, Department of Nursing Midwifery and Health, Northumbria University, Newcastle-upon-Tyne, UK; Lecturer, Department of Nursing Midwifery and Health, Northumbria University, Northumbria University, UK; Lead Nurse for Learning Disabilities, Queen Elizabeth Hospital, Gateshead Health NHS Foundation Trust, UK

**Keywords:** better outcomes, inter-professional learning, mothers with an intellectual disability, student midwives, student nurses

## Abstract

**Aim:**

The aim of this study is to explore the experiences of student learning disability nurses and student midwives in supporting mothers with an intellectual disability.

**Background:**

The Ockenden Report (2022), an independent review into maternity care in the United Kingdom, highlighted workforce challenges and inequalities in maternity services. The report specifically highlighted the need for mothers with intellectual disabilities to have individualised care and support. Public Health England (2021) suggested that continuity of supporting staff is beneficial to mothers with intellectual disabilities. Additionally, specific support is needed for mothers with a learning disability.

**Methods:**

This case study adopted a qualitative approach by interviewing current student learning disability nurses and student midwives through purposive and convenience sampling. Data were gathered by conducting semi-structured interviews. Data was analysed thematically through Braun and Clarke’s (2021) six step process.

**Findings:**

There were three overarching themes identified from the analysed data: Knowledge and Skills; Curriculum, and Exposure and Support when caring for mothers with an intellectual disability.

**Conclusion:**

The findings prompt inter-professional working in maternity services, which in turn can facilitate better outcomes for mothers with an intellectual disability. It may also facilitate the development of materials to raise awareness, reduce stigma, and clarify misconceptions related to supporting mothers with an intellectual disability.

## Introduction

Inter-professional education (IPE) is an educational model whereby healthcare students are trained to integrate their diverse interdisciplinary expertise by learning from, with, and about each other (World Health Organization, 2010). Despite repeated requests over the years to further enhance the student learning experience by extending IPE into practice, it remains typically university based (Barr et al., 2017; O’Leary et al., 2019).

Gaps within professional knowledge around specialist areas have a profound impact upon patients. The aim of this study was to explore the experiences of student nurses and midwives when supporting mothers with an intellectual disability, in order to explore their care and support. The Ockenden Report (2022) highlighted the workforce challenges and inequalities in maternity services for mothers with an intellectual disability. Further to this, Public Health England (2021) also suggested that continuity of supporting staff is beneficial to mothers with intellectual disabilities. It is noted that there are some mentions of supporting disabled patients, but nothing specific to intellectual disabilities. Vernon (2019), suggested creating pathways for women with intellectual disabilities and a hospital passport for use in maternity services. Learning disability and intellectual disability are typically UK and US terms respectively – but are often used interchangeably. Both refer to a global cognitive impairment affecting and individual’s everyday life. In the United Kingdom (UK) the Nursing and Midwifery Council (NMC) maintains separate parts of its professional register: one for Registered Nurses (Learning Disabilty), who are trained to support people with intellectual disabilities, and another for Registered Midwives, who are qualified to provide care throughout pregnancy, childbirth, and the postnatal period.

A literature search was conducted to identify relevant literature on bespoke and interdisciplinary placements within nursing. The search encompassed peer-reviewed papers, policy documents, and grey literature. Search terms were used, including generic terminology related to nursing (e.g. ‘student nurse’, ‘learning disability student nurse’ and ‘healthcare professional’ and specific terminology related to bespoke placements (e.g. ‘bespoke placement’, ‘interdisciplinary placement’ and ‘cross field experience’. Despite an abundance of literature around student nurses and interdisciplinary placements (*n* = 80) covering various topics, only one of the papers reviewed specifically addressed inter-professional student teams (Hood et al., 2022). This gap highlights the lack of published research on evaluating the effectiveness of inter-professional placements for student nurses, despite the ever-growing importance of interprofessional working amongst healthcare professionals.

It is acknowledged that people with learning disabilities have continuously faced barriers when accessing general healthcare services (Walker et al., 2016), and avoidable deaths occur as a result (NHS England & NHS Improvement 2019; Ryan, 2017). However, for mothers-to-be with a learning disability, it is understood that there is an increased likelihood of risk factors and unfavourable outcomes (Fairthorne et al., 2015; Kassee et al., 2023).

There is a notable need for student midwives to have awareness in learning disabilities (Cox et al, 2024), which in turn will facilitate building trust and improved patient outcomes. However, it is acknowledged that there is no such provision (Castell and Kroese, 2016). Similarly, student nurses/learning disabilities do not receive a midwifery clinical placement.

This research was conducted as a case study whereby two student learning disability nurses attended a midwifery placement. Recognising the opportunity to enhance inter-disciplinary education through practical resources, a local healthcare trust (an organisational body within the UK National Health Service responsible for delivering specific healthcare services) agreed to support the students within a midwifery environment. It was therefore possible to evaluate the impact of the bespoke placement via a case study. This evaluation provides valuable insights into the effectiveness and outcomes of the bespoke placement for the students, ultimately contributing to better outcomes for mothers with intellectual disability.

## Methodology

### Design

This study utilised a qualitative design entailing semi-structured interviews to explore the experiences of student learning disability nurses and student midwives. A case study design which involves exploring in-depth an event in its real-life context (Creswell, 2014) was adopted to be able to meet the aims and objectives of the research and to elicit the student experience of supporting mothers with a learning disability.

### Participants and recruitment

Two student learning disability nurses and two student midwives were recruited via purposive and convenience sampling (Campbell et al, 2020). Recruitment involved reaching out to the students via email through university channels. Voluntary participation was invited and interested individuals were provided with consent forms and information sheets detailing the nature of the research.

### Materials

**Figure 1.**
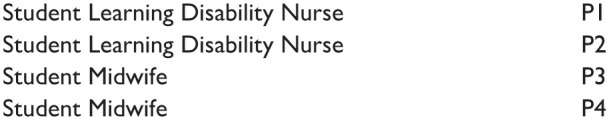
Assignment of unique identifier

Semi-structured interviews were conducted to gather insights into participants’ experiences of midwifery services, learning opportunities, and their perspectives of caring for women with an intellectual disability (interview schedule illustrated in supplementary material). The participants were anonymised and given a numerical identifier. The interviews were audio-recorded with participant consent and transcribed for analysis. Participants were given a unique indentifier to facilitate anonymity; figure 1 illustrates the assignment of the unique identifier.

### Data analysis

The data were analysed using a six-step thematic analysis approach (Braun and Clarke, 2021). The first step involved the researchers familiarising themselves with the transcribed data from the semi-structured interviews.

Coding was analysed line by line to identify meaningful concepts within the data. Following this, all researchers independently reviewed the initial codes for themes which best reflected the participants’ experiences and perspectives. Any minor discrepancies identified between the researchers’, initial coding was identified and resolved, before the identification of themes. Similar codes were grouped together to form the themes.

The researchers then discussed the themes to ensure consistency and accuracy. The themes were examined to determine whether they represented the participants’ perspectives. They were then refined and named concisely to capture the essence of the theme. The final step involved examining the relationships between the themes and subthemes and how they contributed to the overall understanding of the research topic. Patterns identified between the themes were discussed.

### Ethical considerations

Ethical approval was sought and granted from the university ethics committee, project ID number 3881. All ethical guidelines were adhered to including, confidentiality, informed consent and data protection. Participants were not studying modules connected to the researchers; therefore, there were no conflicts of interest or possibility of coercion to participate.

Participants were provided with a full participant information sheet, consent forms and an opportunity to attend and evaluate a new bespoke placement, to further enhance their clinical practice.

## Results

Three main themes were identified with 14 corresponding subthemes (See Table 1). The three main themes were: Knowledge and skills, Curriculum, and Exposure and support when caring for mothers with an intellectual disability.

**Table 1.** Themes and subthemes identified from participant interviews.

Themes	Subthemes
Knowledge and skills	Language
	Lack of knowledge Specialist knowledge of intellectual disabilities skills
Curriculum	Placement learning Curriculum content
Exposure and support when caring for mothers with a learning disability	Exposure to mothers with intellectual disability Support Reasonable adjustment Liaison nurse Safeguarding Working together

### Theme 1 – Knowledge and Skills

The first theme is essential in understanding how the knowledge of intellectual disabilities, the language used within midwifery services, and the clinical skills acquired within midwifery services can influence the perspectives of the students. By exploring their experiences, valuable insights were gained into how the knowledge of learning disability nurses can be optimised within midwifery services to enhance the care of women with intellectual disabilities from the antenatal through to postnatal period.

#### Language

The students spoke about the language differences between the two healthcare professions and how whilst the same terms were used, they had very different meanings, highlighting the differences between medical terminology amongst professionals: ‘*when they said all they've been sectioned I would instantly think of the Mental Health Act, they were thinking of caesarean section so there was two totally very separate meanings’ (P1).*

Additionally, the students described how terminology used by their own specific professions becomes ‘the norm’ for them but can be difficult for others to express:

‘*. . . some people were quite scared to ask the question, have you ever been told you have LD [learning disability] or been diagnosed as having LD? there’s still a little bit of a taboo around asking those questions whereas learning disability nurses, that’s kind of what we do daily and it doesn’t come naturally to other professions. I understand that . . . if they are educated then that’s the key to improve the service’ (P1).*

They highlighted the difficulty professionals may have in asking if someone has an intellectual disability, suggesting that education around caring for this population, may encourage and support staff to feel comfortable discussing their needs.

#### Lack of knowledge

It was highlighted that there was a lack of awareness around learning disability nurses’ availability within maternity settings and referral pathways: *‘I did not know that there was anyone that we could go to specifically for support, for people with learning disabilities. I mean, that’s not to say that they’re not there. I just think they’re not as well advertised’*, (P3). The participants seemed to suggest that there was a lack of knowledge transparency with referral pathways, which may impact the care given within maternity services. Furthermore, the students highlighted a lack of knowledge around what learning disability nurses do and how this knowledge could be used to enhance the patient experience. They implied that facilitating a familiarity with the profession and the services they provide may lead to higher levels of support and collaboration: ‘*So, I think raising knowledge is definitely going to help a lot of people because if you don’t know that this service exists, you’re not going to know that you can send people that support’ (P3)*. Additionally, without intellectual disability support, it was difficult to fully care for women with intellectual disabilities and emotionally impacted the healthcare professionals as well as the woman: *‘the thing that upset me about that was that I couldn’t tell her specifically what was going to happen when she’d got there because I didn’t know what would happen when she got there’, (P3)*.

#### Specialist learning disability knowledge

Furthermore, they spoke about the specialist knowledge that learning disability nurses have which can positively impact upon mothers with an intellectual disability in a midwifery setting: ‘*Explaining what I did as a student LD nurse and trying to change some of those attitudes and misconceptions around mothers with LD and actually mental health conditions as well and that kind of came out a lot’ (P1)*, and how the specialist knowledge could change the experience for birthing mothers, resulting in more positive outcomes for mother and baby. The participants discussed how safeguarding referrals were often made once health professionals knew the mother had an intellectual disability, but with more knowledge and specialist support, these mothers may be able to sufficiently care for their babies, rather than having them removed from their care.

*‘There’s a reason why learning disability nurses are really important in this role particularly in relation to the percentage of mothers (with learning disabilities) whose babies get adopted/fostered/in the care system and you know we know that evidence is quite high which is really sad so it’s just making sure that we share that knowledge’, (P1).*

#### Skills

The bespoke placement identified the enhancement of skills for both the student learning disability nurses and the student midwives: *‘They taught me a lot so that was helpful for later in terms of my own development, and if I was ever to support a woman with LD in the future who was pregnant’ (P1).* However, it was felt that the skills were not fully collaborative, and there was a ‘disconnect’ between the professions: ‘*There was still a little bit I would say of a disconnect in terms of both fields have solely totally different sets of skills, yet we have shared skills and those skills and knowledge for each field’, (P1).* This reinforced the notion that IPE is an important element of the curriculum and should be embedded into universities to foster relationships and understanding at the earliest point.

For the student midwives, there was the doubt that they lacked the skills to fully recognise and appropriately treat a woman with an intellectual disability, highlighting how they had not had any education around working clinically with these women:

*‘No one has had that practice, a lot of people haven’t got the clinical skills. They are going out and a lot of people haven’t got that kind of bedside manner, and you don’t want to say the wrong kind of thing and you know . . . I shouldn't have said that kind of thing, has that come across wrong? You want to treat everybody the same, but you want to make sure that there is still support there as well, you are not ignoring the disability’ (P4).*

Furthermore, they felt this was extended beyond the student years into qualified midwifery practice:

‘*. . . and I have spoken to the midwives, and they don’t have the proper training, they feel like they haven’t got enough training for it, and there’s not enough, there's like a lack of specific resources, like your diagrams and stuff like that. Anything that they need, like pictures or anything, there is not enough stuff like that’ (P4).*

### Theme 2 – Curriculum

The second theme highlights the gap within the nursing and midwifery curriculum around inter-professional learning as well as how beneficial bespoke placements such as this pilot are to their learning experience. By exploring their experiences, further insights were gained into how the curriculum can be optimised to enhance the learning journey and inter-professional skills of students.

#### Placement learning

Participants commented on how they viewed the placement experience in the midwifery ward: *‘I think it would be really really beneficial for student LD nurses to experience ante and post-natal care. . .’* (P1); ‘*I personally found the placement really helpful because I got to learn quite a lot of skills’ . . .* ‘*I definitely think that they should continue to happen’* (P2); ‘*I think sending the LD nurses into the ward would be really beneficial because I probably could count on my one hand, how many people have a support system in-place’* (P3). They highlighted the benefits of the placement, for both student nurses and student midwives, detailing skill enhancement and collaborative practices as important concepts they would not have gained elsewhere.

Additionally, participants reported this as important for student midwives in their placement experience:

*‘I also think for student midwives experience day service or day centre or within the peri natal health team yes, it might be more mental health based but would still get women with LD coming through the service and obviously those link quite closely together; and also knowing who to contact for support and advice the area’* (P1); ‘*. . .all midwives should have a place in LD’* (P2);‘. . .*even if it was one/two days’* (P4).

There was a degree of peer support amongst the students whilst on the placement, particularly between the Learning Disability students; ‘*I did peer support with another LD nursing student, we would reflect together would have a bit of a debrief . . . both in the same situation of being quite unfamiliar with the environment . . . the processes particularly on the post natal ward is a locked ward but obviously we have experience with but it's a totally different set up to what we are used to’* (P1).

#### Curriculum content

When asked about learning disability nursing and midwifery, students learning together participants spoke about the curricula, specifically commenting on where they saw the gaps in content. There was a strong theme of embedding further inter-professional experiences within the curriculum to enhance their knowledge and skill development: ‘*Not in the curriculum it was mainly other activities’* (P1) ‘*I don’t recall doing that with any student midwives. . .’ (*P1); ‘*inter-professional learning we do, I think it’s once a year, and we do a kind of MDT practice meeting, other than that not much’* (P3); ‘*I don’t think we’ve had very much official at university. I think I can remember one lecture . . . in second year’* (P3); ‘*There’s nothing, no, there wasn’t anything specific to LD. Not at all . . . There is definitely not enough’* (P4).

The participants highlighted several reports and guidance policies which detail the importance of shared education, commenting upon how IPE would be beneficial to minimise risk around health outcomes; *‘I think in terms of formalised shared learning I think that would be really beneficial . . . the public health guidance . . . all of the reports that have come out recently in terms of papers and government guidance . . . there’s a lot of need for it . . . shared education around safeguarding’* (P1).

Participants spoke of future possibilities of how inter-professional learning could be enhanced within the curriculum: ‘*there could be a whole module done on maternity care and learning disabilities we can draw in mental health you could then draw in adult nursing students who may be working in A and E [Accident and Emergency]and present with a woman who has LD and gone into labour. . .’* (P1).

*‘Even if it was simulation and as well to appreciate what each other's roles are because I still think there is a little bit of a divide in terms of some people are unsure what the others role is. I think that just comes with time and experience. Even if that was done in first year in inter-professional learning week I know it encompasses all fields of nursing but if midwifery was in there they might do different things’ (P1).*

They highlighted the need for ongoing IPE throughout their degree to ensure the right level of knowledge to take forward into clinical practice: ‘*If we had some more of that in third year . . . it would be beneficial because it would be fresh in our head and we would know that that’s something to take forward with us as qualified midwives . . .’ (P3).* *‘100% definitely, it should be part of one of the modules, like in first year, well every year, but first year definitely’ (*P4).

### Theme 3 Exposure and support when caring for mothers with a learning disability

The third and final theme encapsulated the exposure to, and care of, a woman with intellectual disabilities within the midwifery setting. This theme was essential in understanding the levels of exposure to women with intellectual disabilities and the roles of specialist liaison nurses to ensure appropriate safeguarding provisions were made and the correct reasonable adjustments and support are provided.

#### Exposure to mothers with learning disabilities

When asked about supporting mothers with intellectual disabilities P2 reported only having supported patients with parents having intellectual disabilities, whereas P3 said she had supported two mothers, one in community and the other in the ante-natal ward. They felt that a lack of experience impacted their confidence to care for women with intellectual disabilities. P3 furthered this by saying ‘I *don’t feel confident as a student . . . once I’m a qualified midwife. . . but probably because I haven’t experienced it much . . . to gain confidence you need that experience and exposure’*. P4 said ‘*I have only experienced women with LD in community placement . . . I haven’t had to care for anybody like that . . . I really wouldn’t know the road to go down for extra support. . .’*

#### Support

Support needed for mothers with intellectual disabilities was referred to as an important matter, linked to raising awareness. The participants felt greater awareness was needed around how to support women with intellectual disabilities, as well as knowledge of services to collaborate within their care: ‘*it’s definitely raising awareness . . . it’s definitely going to help a lot of people. . .if you don’t know what the service is you’re not going to know that you can send people to that support*’ (P3). Additionally, ‘. . .*a meeting between the multi-disciplinary team. . .and saying right now where is the care for this woman . . . is someone going to be there for her and her baby. . .discharged from hospital then what after day 10. . .’ (P4).*

#### Reasonable adjustments

The subject of reasonable adjustments was raised by all participants. The student learning disability nurses emphasised the importance of reasonable adjustments when caring for a woman with an intellectual disability, highlighting the lack of understanding from other professionals.*‘They didn’t make reasonable adjustments. . .perhaps there was a lack of understanding of why somebody needed an adjustment’* (P1). Furthering the point by outlining what that might be:

*‘a LD nurse might see a reasonable adjustment in information explained a particular way . . . someone else might say that woman can’t bond with her child or parent successfully . . . when we know that’s not the case . . . there is still a way to go in terms of understanding about reasonable adjustments . . . constipation is a big killer . . . the LeDer report says LD nurses pick this up instantly, not all midwives understood the impact of analgesia. . .*’ (P1).

‘*Making sure people understood what a reasonable adjustment was, and what a learning disability was . . . not just mothers having babies with Downs Syndrome, in maternity services they think of the baby rather than the mother’* (P1).

Awareness and understanding of reasonable adjustment are needed through: ‘*training in hospitals’ (*P1), as well as the inconsistency in hospital records, ‘*some hospitals do have flags for women with LD some don’t, it might be at the bottom of the page’ (*P1).

When discussing reasonable adjustments further, P2 said she would give guidance to see what reasonable adjustments to use to *‘keep everyone in the loop . . . then look at what reasonable adjustments are . . . like hospital passports, times of appointments . . . time for people to talk and access the bus’.*

Low uptake of attending appointments was noted: ‘. . .*it needs highlighting there are a lot of missed appointments for people with learning disabilities who are pregnant . . . a lot to do with mistrust in the system . . . scared of being treated differently . . . that the baby’s going to be taken off them*’ (P2).

Having a baby is not straightforward, participants considered challenges faced by the non-learning-disabled population and how this is amplified for those with intellectual disability: ‘*I would find having a baby terrifying and I don’t have a LD’*, (P2). They highlighted what reasonable adjustments could include, such as *‘having someone there, as well as someone who can communicate . . . in a way they can understand, making aids . . . notice boards . . . making sure they understand what is expected of them. . .ask the woman if they require any further support’ (*P3). An individualised approach in the sense of: ‘*asking people on an individual basis what support we can provide . . . not everyone is going to require the same support’* (P3).

Support from community midwives was acknowledged by P4: ‘. . .*they were really good at getting extra stuff and asking all the time if she needed anything . . . making sure she had good support network’*. Whilst on a community placement she recounted the reasonable adjustments that were made: ‘. . . (she was) *shown how to make a bottle because she didn’t want to breastfeed . . . (they) printed out the list for hospital . . . obviously hers wasn’t so bad because she could read a little bit’* (P4).

#### Liaison nurse

LD student nurse participants spoke positively of the hospital Liaison Nurse who specialises in suppoting patients with intellectual disabilities: ‘*We had support from the hospital Liaison Nurse for Learning Disabilities who was fantastic for students, but also bridging the gap between maternity and LD’* (P1): ‘*There was some good work with the LD Liaison Nurse, including posters up’ (*P2).The student midwives, however, did not know such a role existed to advocate and support mothers with a learning disability: ‘*I didn’t know there was a liaison LD nurse available . . . now I do I will find out more, and I know where to go’* (P3).*‘I know now that you can enlist the help of a LD nurse if you had a woman with LD giving birth. . .I’ve never been told that’* (P4).

#### Safeguarding

The issue of safeguarding was recounted by participants: ‘*what does and doesn’t constitute a safeguarding is an issue particularly exploring the experiences of mothers with LD in maternity services’* (P2). They noted how staff in the midwifery area were noted to refer to safeguarding when a mother presented with an intellectual disability, believing this is to be a lack of awareness around the capabilities of these women: ‘*there was a lack of awareness . . . LD nurses would look and go that’s not a safeguarding (concern) they need some further education. . .’* (P1).

The participants felt that the mothers weren’t ‘trusted’ to look after children if they had an intellectual disability: ‘*. . .It felt like she was being treated differently, that she wasn’t trusted. . .wasn’t capable of looking after a baby’* (P2).

#### Working together

The participants highlighted a lack of awareness around working together collaboratively across services to support women with intellectual disabilities: ‘*I’ve never come across LD nurses with the midwives in any areas of my placement, to be honest – and again, I didn't actually know that there were people that actually worked together because it’s not anything I've come across’ (P3).*They emphasised the importance of collaboration and contacting those specialist services, to provide quality care to women with intellectual disabilities: ‘*It’s important to just continue to collaborate . . . they were trying to collaborate more, really on board with student LD nurses coming on the ward . . . but not sure how often they would contact LD support when they have somebody with LD’ (P*2).

## Discussion

The key issues affecting women with intellectual disabilities within maternity care arises from their greater levels of co-morbidity, possible compromised ability to communicate their needs, and the limited research on best practice in this area (Mueller et al., 2019). This study has provided insights into the impact of specialist placements for inter-professional nurse education. It revealed several examples of how multiple interpretations of terminology can lead to miscommunication with inter-professional teams. The language used in differing healthcare professions can cause confusion and affect communication across specialisms (Jayatilake and Oyibo, 2023). Sherriff et al. (2017) suggested that the use of medical terminology across departments varies and that standardised terminology would significantly enhance communication and cross disciplinary understanding.

There also appeared to be a lack of knowledge around what learning disability nurses were and what they could offer within a midwifery setting. Involving learning disability nurses allowed for challenges associated with communication or behaviour to be addressed thus reducing diagnostic overshadowing - a clinical bias in which a healthcare professional incorrectly attributes a person’s new or unrelated symptoms to an existing condition, most often a learning disability, mental health condition, or autism—resulting in missed, delayed, or inadequate diagnosis and treatment (Manohar et al., 2016). As evidenced in this case-study, a lack of cross disciplinary knowledge of learning disability nursing, and therefore a lack of specialist input with regarding to learning disability practice, has the potential to result in adverse outcomes for the patient (Jaques et al., 2018). This included failure to fully understand patient needs or making inappropriate safeguarding referrals.

This study offers promising evidence of the effectiveness of learning disability nurses within midwifery settings and the specialist knowledge they can bring to supporting women with an intellectual disability. Unique in its approach, the study captures the perspectives of both student learning disability nurses and student midwives directly involved in caring for women with intellectual disabilities during pregnancy and birth.

The study found that having a placement in midwifery services was valuable for student learning disability nurses. Similarly, it was highlighted that student midwives would benefit from a placement within mental health and intellectual disability services. By extending the learning from theory, learning disability and midwifery students can learn together in a clinical placement. Peer support was also found to be valuable throughout the placement amongst students who had no previous midwifery experience, thus gravitating to other learners in the same situation. This approach can build on previous research which highlights the notion that midwives lack confidence to support people with learning disabilities and co-produced learning disability awareness training is crucial to promoting personalised maternity care (Cox et al., 2024). Moreover, implementation of the Oliver McGowan Training as mandatory would help to address the issue. The Oliver McGowan Training (formally The Oliver McGowan Mandatory Training on Learning Disability and Autism) is the standardised, nationwide training for all health and social care staff in England on how to better support people with learning disabilities and autistic people. The training was created in response to the death of Oliver McGowan, a teenager with autism and a mild learning disability who died in 2016 after being given antipsychotic medication against his and his parents’ wishes.The LeDeR review into his death found failures in understanding autism, communication needs, and reasonable adjustments, and recommended mandatory training for all staff (NHS England & NHS Improvement 2020). This campaign became known as “Oliver’s Campaign” and led to a major policy change. Under the Health and Care at 2022 this training is now mandatory for all NHS and Social Care staff.

The importance of training in supporting people with LD in health and social care delivered by Experts by Experience is noteworthy and highlighted by Ridley and Flynn (2024). They emphasise that training delivered by Experts by Experience offers a deeper, more authentic understanding of the realities faced by people with learning disabilities in health and social care. They argue that this approach helps staff challenge assumptions, improve communication, and provide safer, more person-centred support.

It was noted that there was limited theoretical content in the current midwifery curricula on the subject of intellectual disabilities. Equally, it was highlighted that any IPE content did not translate to student learning disability nurses and student midwives learning together. Suggestions were given by participants of how this should be operationalised. This included a continuum of activities at year one to three, ranging from IPE workshops, simulated learning, to an entire module. Simulated reality learning packages have been found to be beneficial for all nursing and midwifery students to improve confidence, knowledge, and decision-making (Saunder and Berridge, 2015). Through ongoing joint simulated practice across the curriculum students could develop their knowledge and skills throughout their programme of study.

Participant responses varied on their degree of exposure to pregnant women with intellectual disabilities. Evidence suggested that mothers with an intellectual disability were more likely to disengage with maternity services, have limited access to the full provision of maternity services and unmet needs during pregnancy (Daniels and Douglass, 2021). Whilst there is guidance from Public Health England (2021) to support mothers with intellectual disabilities, up to 50% may have their child removed (Gould and Dodd, 2014). The findings from this research echo the evidence and currently held views; thus, a greater exposure and experience of supporting mothers with intellectual disability is needed from novice to expert level within the professions.

Reasonable adjustments are a legal requirement within the remit of the Equality Act (2010) and was the most significant issue within theme 3. Reasonable adjustments in a hospital setting are changes or adaptations made to remove barriers so that people with disabilities can access healthcare safely, effectively, and on an equal basis with others. This may include things like longer appointment times, quiet waiting areas, communication support, personalised care plans, or allowing carers to stay - all tailored to the person’s individual needs. Participants identified a lack of understanding of what a reasonable adjustment was and furthers the notion of ill-prepared healthcare professionals resulting in negative experiences of women with intellectual disabilities in maternity services (Dowling et al., 2023). It was evident in the data that there were vast differences in interventions, from implementing reasonable adjustments to a referral to the safeguarding team. The former being a supportive intervention, the latter may result in more negative outcomes for mother and infant prior to support being implemented. There is a greater need for awareness raising for healthcare professionals and is crucial to adequately and safely, support mothers with intellectual disabilities in maternity services in a person-centred manner and with a holistic approach.

The Learning Disability Liaison Nurse was described as a positive influence for both the students on the placement and the mothers with Intellectual Disabilities. A Learning Disability Liaison Nurse is a specialist nurse in hospitals who supports people with learning disabilities to access safe, effective, and equitable healthcare. They help staff make reasonable adjustments, improve communication, coordinate care, advocate for the patient’s needs, and ensure that treatment plans are understood and followed, reducing avoidable health inequalities. NHS England (2023) encourages trusts to have this specialist support, but it is not mandated in legislation and therefore provision is varied across the nation. The Learning Disability liaison nurse can increase the confidence of staff without intellectual disability experience and ensures compliance with legislation, including capacity and safeguarding (MacArthur et al., 2015). However, there was a distinct lack of knowledge around the role from the midwifery perspective. With greater awareness of the specialist roles, collaborations between specialist intellectual disability services and other healthcare professionals can offer access to practitioners with the knowledge and expertise to address the challenges they may experience (Heslop et al., 2013).

This case study addresses a critical gap in knowledge, providing valuable insights into the student nurse curriculum and placement learning opportunities. However, the small sample size of two Learning Disability student nurses and two midwifery students may not fully capture the potential impact it may have on IPE. Whilst the evidence is evolving, larger-scale placements, detailed curricula content and further research studies would offer a more comprehensive understanding.

## Conclusion

This study demonstrates the value of inter-professional learning between student Learning Disability nurses and student midwives, showing how a bespoke midwifery placement can enhance skills, confidence, and collaboration when supporting mothers with intellectual disabilities. Gaps in curricula, limited clinical exposure, and a lack of awareness of specialist LD roles contribute to uncertainty around reasonable adjustments and safeguarding, which can negatively affect care. The placement helped students understand each other’s roles, challenge misconceptions, and strengthen communication-key to reducing diagnostic overshadowing and promoting person-centred maternity care. The LD Liaison Nurse was identified as an important source of specialist support, though provision remains inconsistent nationally. While the small sample restricts transferability, the study highlights the need for integrated IPE, improved training, and expanded placement opportunities. Further research could explore the views of midwifery staff or the patients’ perspective, offering deeper insight into how maternity services can better support women with intellectual disabilities.

**Key points for policy, practice and research**

- This case study demonstrates how collaboration between Learning Disability (LD) nurses and midwives can improve communication, reduce diagnostic overshadowing, and enhance person-centred care. Embedding structured inter-professional learning opportunities may support better outcomes for mothers with intellectual disabilities.
- Greater cross-disciplinary understanding can promote more consistent use of reasonable adjustments, including accessible communication, tailored appointment structures, and clearer care pathways – reducing inequities faced by mothers with intellectual disabilities.
- Findings indicate a need for resources that raise awareness, reduce stigma, and challenge misconceptions about intellectual disability. Co-produced materials and training, including contributions from Experts by Experience, could enhance confidence and competence in maternity teams.
- Universities may consider integrating ongoing inter-professional education, simulated learning, and cross-field placements to ensure students acquire the knowledge and skills necessary to support this population effectively.
- Improved national awareness and more consistent provision of LD Liaison Nurses could strengthen specialist input, safeguarding decisions, and multidisciplinary collaboration in maternity services.
- Larger-scale studies are needed to evaluate the wider impact of inter-professional placements. Further research should also explore the perspectives of midwifery staff and mothers with intellectual disabilities to inform service improvements and policy development.

## Appendix

**Appendix 1.** Interview schedule.

Topic area	Questions	Probes
Experience of learning disability students in a midwifery placement	What is your experience of student learning disability nurses in MW placement?	• Have you ever worked with a student LD Nurse? • Have you ever worked with a MW student? • Have you had a clinical placement in MW?
Experience of LD and MW working together	What is your experience of Learning Disability Nurses and Midwives working together?	• What is your general experience of LD and MW working together? • Have you ever come across this? • Have you ever tried to enlist the help of a LD Nurse or MW?
Experience of LD and MW learning together	What is your experience of inter-professional learning, with student Learning Disability Nurses and Midwives?	• Have you ever been in a seminar or teaching activity on campus with LD Student Nurse or a student MW? • Have you ever attended a learning opportunity in clinical placement with a Student LD Nurse or student MW?
Supporting a mother with a learning disability	Can you tell me about your understanding of supporting a mother with a learning disability in midwifery services?	• Is this something you have come across? • Is this an area of your practice that you are aware of ? • Are you confident to do this?
Future placements	Can you describe suggestions for future placements for LD student nurses and student midwives?	• What are your thoughts about this? • Is it something that you feel should take place? • Is this an area of learning that could be explored in further detail?
